# Ethical issues in multi-agent AI systems for healthcare: a narrative review

**DOI:** 10.3389/fpubh.2026.1792627

**Published:** 2026-04-28

**Authors:** Zhibin Xie, Hongyu Wang, Lexuan Dai, Zikai Wang, Haitao Song, Jingzhe Qian

**Affiliations:** 1 KoGuan School of Law, Shanghai Jiao Tong University, Shanghai, China; 2 The Chinese University of Hong Kong, Hong Kong, Hong Kong SAR, China; 3 University of Southern California, Los Angeles, CA, United States; 4 Shanghai Artificial Intelligence Research Institute, Shanghai, China

**Keywords:** agentic AI systems, autonomous decision-making, digital health, healthcare ethics, responsibility and accountability

## Abstract

**Introduction:**

Multi-agent AI systems are believed to bring significant improvements in digital health, but it also brings new and more serious ethical issues. Such systems distribute the decision-making process among multiple interacting agents, and this decentralized decision-making system has raised ethical concerns in the medical field. On the one hand, it continues the ethical issues of traditional AI tools; on the other hand, the interaction processes within complex systems have also brought about new dilemmas. This narrative review aims to synthesize the ethical issues related to multi-agent AI systems in healthcare presented and explore the corresponding mitigation strategies.

**Methods:**

The study outcomes were synthesized using a narrative approach. Relevant records were gathered through Boolean searches in databases such as PubMed, Scopus, and Web of Science. A total of 21 articles related to multi-agent AI, healthcare, and ethical issues are included in this review.

**Results:**

Seven key ethical challenges were identified: (1) compound opacity, where interacting AI agents create layers of inscrutable decision-making; (2) error propagation and attribution difficulties, complicating accountability for clinical harm; (3) increased clinician dependence and automation bias, leading to potential deskilling and overreliance; (4) erosion of human oversight, as multi-agent AI systems operate beyond effective human control; (5) privacy and data security risks, stemming from complex data flows among agents; (6) threats to patient autonomy and informed consent, due to opaque or paternalistic AI recommendations; and (7) contextual blindness, reflecting a loss of individualized patient understanding in modular AI workflows. Furthermore, this review also summarized solutions proposed in the existing literature for these ethical issues.

**Conclusions:**

Multi-agent AI systems intensify existing ethical concerns in healthcare by distributing decision-making and blurring responsibility. To mitigate these issues, recent research advocates for the development of adaptive governance models, clear accountability frameworks, human–AI collaboration structures that preserve clinician authority, enhanced systems for explainability, and privacy-centered designs. In order to successfully incorporate agentic AI into healthcare, it is essential to maintain transparency, protect patient rights, and ensure that human-centered values continue to guide clinical decision-making in an era dominated by autonomous, interacting AI systems.

## 1 Introduction

Artificial intelligence (AI) is playing an increasingly prominent role in healthcare, from diagnostic image analysis to personalized treatment recommendations (1, 2). Recent advances have given rise to autonomous agents and multi-agent systems (MAS) architectures in clinical AI (3, 4). To facilitate the subsequent analysis, it is essential to distinguish between “Agentic AI” and “Multi-Agent Systems” (MAS). “Agentic AI” refers to the functional capability of a system to exhibit goal-directed autonomy—perceiving its environment, reasoning about objectives, and executing actions without continuous human prompting (5, 6). In contrast, “MAS” refers to an architectural framework where decision-making is distributed across multiple interacting entities. While distinct, these concepts are increasingly convergent in healthcare applications (7). Consequently, this review focuses on “multi-agent AI systems”—defined here as systems that are both structurally distributed and functionally agentic (6)—laying the groundwork for understanding the complex ethical landscape they create.

Unlike a single algorithm performing a narrow task, multi-agent AI systems involve multiple AI agents—often powered by large language models (LLMs) and other specialized models—interacting and collaborating with minimal human intervention (8–10). The integration of multi-agent AI systems into clinical practice presents a paradigm shift, moving beyond individual AI tools into a more interconnected, agent-based approach. Such systems can potentially manage complex, dynamic tasks in a way that individual models cannot, by simultaneously processing data from diverse sources, generating recommendations, and executing tasks (11, 12). In practice, this means that multi-agent AI could revolutionize care delivery, from triaging patients to managing entire clinical workflows autonomously (13). For example, multi-agent AI systems have been employed in intensive care, perioperative management, radiology, and medical coding, offering improved workflow efficiency, personalized care, and enhanced patient safety (14–17). These innovations promise not only to increase efficiency but also to reduce human error, optimize resource allocation, and improve patient outcomes in an era marked by rising demand for healthcare services (13). This shift from stand-alone decision support tools to agentic AI systems offers major potential for public health efficiency and equity,.

However, these advances also inherit longstanding ethical concerns of earlier AI and in many cases amplify them (15). Issues such as opacity (the “black-box” nature of complex models), algorithmic bias, and even generative AI hallucinations (AI confidently producing false information) are well-documented challenges in medical AI (18–20). Multi-agent architectures risk compounding these problems. Each agent in a chain may be a black-box itself, and their interactions can create layers of inscrutability beyond what a single model would produce (21, 22). Biases present in one component can cascade through others, making errors harder to detect and correct (23). Recent studies identified core challenges of current LLM-based AI—security, privacy, bias, misinformation, high energy use, lack of transparency, etc.—and warned that these “can propagate into Agentic AI” (24, 25). In other words, a multi-agent healthcare system not only carries the sum of its parts' risks, but introduces new ethical and safety challenges by virtue of complex agent interactions.

Beyond the ethical challenges inherited from earlier AI iterations, agentic AI in healthcare introduces a distinct set of concerns, notably opaque decision-making, constrained human oversight, complexities in multi-agent coordination, and the critical issue of ambiguous ethical accountability when autonomous agents undertake clinical decisions (26, 27). When multiple autonomous agents collaborate to analyze data, make recommendations, or guide care pathways, transparency becomes harder to achieve and responsibility harder to assign because each agent's role in arriving at a final outcome is intertwined with others (6). In traditional clinical practice and even in earlier AI deployments, accountability frameworks typically presuppose that responsibility can be traced back to a specific individual, professional, or well-defined tool. However, in MAS, decision-making authority is distributed across a network of interacting AI modules and human participants, creating a situation in which no single actor fully controls or comprehends the system's outputs (28). This distributed structure weakens established mechanisms of professional and legal accountability and challenges core ethical principles such as non-maleficence and informed consent, because patients and practitioners alike may not be able to interpret or contest how an autonomous recommendation was reached (20, 29). This uncertainty not only erodes trust in healthcare but also compromises patient safety and weakens professional accountability, emphasizing the critical need for clear ethical guidelines that can address who is responsible in these complex systems—a challenge that remains insufficiently addressed in current regulatory frameworks (30, 31).

This narrative review investigates the evolving ethical considerations associated with multi-agent AI systems in healthcare, concentrating on the allocation and potential misallocation of responsibility within these intricate systems. Through a synthesis of recent literature spanning from 2022 to 2025, we delineate the principal challenges, such as compound opacity, cascading errors, over-reliance by clinicians, erosion of human oversight, privacy risks, threats to patient autonomy, and contextual insensitivity. In addition, We discuss proposed mitigation strategies and design principles to preserve ethical norms (transparency, accountability, and patient rights) in the face of these challenges. Ultimately, as multi-agent AI begins to “act” within clinical environments, the medical community must proactively address who is answerable when things go wrong and how to maintain human-centered care in an era of agentic AI.

## 2 Methods

### 2.1 Review design and aims

This narrative review was designed to synthesize and contextualize the ethical challenges posed by multi-agent AI systems in healthcare, as well as to explore the mitigation strategies proposed for those challenges. We chose a narrative (qualitative) review approach because the topic is emerging and involves diverse types of evidence (technical reports, conceptual analyses, and empirical studies), making a traditional systematic review impractical. This approach allowed us to capture a broad spectrum of issues and insights. The importance of this review is rooted in the novelty of multi-agent AI in healthcare and the lack of consolidated ethical guidance in this area – addressing this gap benefits researchers, clinicians, and policymakers by highlighting key unresolved ethical questions. The aim of the review is explicitly to identify the range of ethical issues associated with multi-agent AI systems in clinical settings and to summarize any recommended solutions, thereby guiding future development and governance of such technologies.

### 2.2 Search strategy

A comprehensive literature search was conducted to identify relevant articles and reviews on the ethical implications of multi-agent AI systems in healthcare. This search was conducted in three major databases (PubMed, Scopus, and Web of Science), with the search period limited to English studies published from January 2022 to December 2025. The search strategy, including all search strings, was defined a priori before the literature search was conducted. As shown in Table 1, structured combinations of keywords covering three domains (multi-agent AI, healthcare, and ethical issues) were applied consistently across all databases. This time frame coincides with the explosive growth of research on autonomous AI agents and LLMs, aligning with the purpose of this study.

**Table 1:** Database search strategy.

Database	Search string
PubMED	“(“multi-agent system*“ OR “multiagent system*“ OR “agent-based system*“ OR “autonomous agent*“ OR “AI agent*“) AND (“medical“ OR “healthcare“ OR “clinical“ OR “biomedical“) AND (“liability“ OR “responsibility“ OR “accountability“ OR “ethic*“)“
Scopus	TITLE-ABS-KEY( (“multi-agent system*“ OR “multiagent system*“ OR “agent-based system*“ OR “autonomous agent*“ OR “AI agent*“) AND (“medical“ OR “healthcare“ OR “clinical“ OR “biomedical“) AND (“liability“ OR “responsibility“ OR “accountability“ OR “ethic*“) )
Web of Science	TS=("multi-agent system*" OR "multiagent system*" OR "agent-based system*" OR "autonomous agent*" OR "AI agent*") AND ("medical" OR "healthcare" OR "clinical" OR "biomedical") AND ("liability" OR "responsibility" OR "accountability" OR "ethic*")

### 2.3 Eligibility criteria and study selection process

The researchers conducted an initial screening based on the titles and abstracts of the studies. For studies that passed the initial screening, full texts were retrieved for further assessment against the eligibility criteria.

The inclusion criteria for this review focused on studies that addressed multi-agent and/or agentic AI systems specifically within healthcare contexts, with a particular emphasis on ethical, governance, and accountability-related dimensions of these technologies. Studies were included when they explicitly described or implied multi-agent or agentic characteristics in healthcare AI systems, thereby ensuring conceptual consistency across the analyzed literature. Eligible articles included peer-reviewed research, systematic reviews, and expert commentaries published in English between 2022 and 2025.

Studies were excluded if they focused on AI applications outside healthcare (e.g., finance, robotics, or other non-medical domains), or if they were purely technical and did not engage with ethical, governance, or societal implications of AI systems. Studies that discussed AI ethics in healthcare but did not address multi-agent or interactive system dynamics were also excluded. Non-English publications were not considered.

### 2.4 Data analysis and limitations

Data analysis employed a narrative and thematic synthesis methodology,adhering to the SANRA guidelines for narrative reviews (32). After initial database searches, articles were screened by title and abstract, followed by full-text assessment to confirm eligibility. Key information was extracted from each included study, focusing on ethical challenges in multi-agent AI systems and any proposed mitigation strategies.

Instead of forcing the data into predefined categories, we took an inductive approach, allowing themes to emerge naturally from the texts. To ensure reliability, the authors first reviewed the material independently. We then met to compare our findings, discussing any differences in interpretation until we reached a consensus.

Finally, we clustered the overlapping and frequently cited issues into broader themes. After several rounds of refinement, these themes became the structural foundation for the Results section. Because the extracted data was inherently qualitative, we focused entirely on building a well-cited narrative synthesis rather than attempting a quantitative analysis. This approach allowed us to capture the complexity and nuance of the ethical issues while also providing a clear and organized presentation of the findings.

Despite the comprehensiveness of the search strategy, several limitations should be acknowledged. This review was restricted to English-language publications published between 2022 and 2025, which may have resulted in the exclusion of relevant studies in other languages as well as studies published outside the predefined time frame, thereby limiting the global and temporal scope of the analysis. Besides, given the rapidly evolving nature of AI technologies and their ethical implications in healthcare, some relevant studies may still have been overlooked.

In addition, as a narrative review, this study relies on interpretive synthesis, which introduces an element of subjectivity. Although we aimed to provide a balanced account of the key ethical issues, study selection, thematic grouping, and analytical emphasis may have been influenced by the authors' perspectives. Compared to quantitative evidence syntheses such as meta-analyses, this approach may carry a higher risk of interpretive bias.

## 3 Results

**Figure 1:**
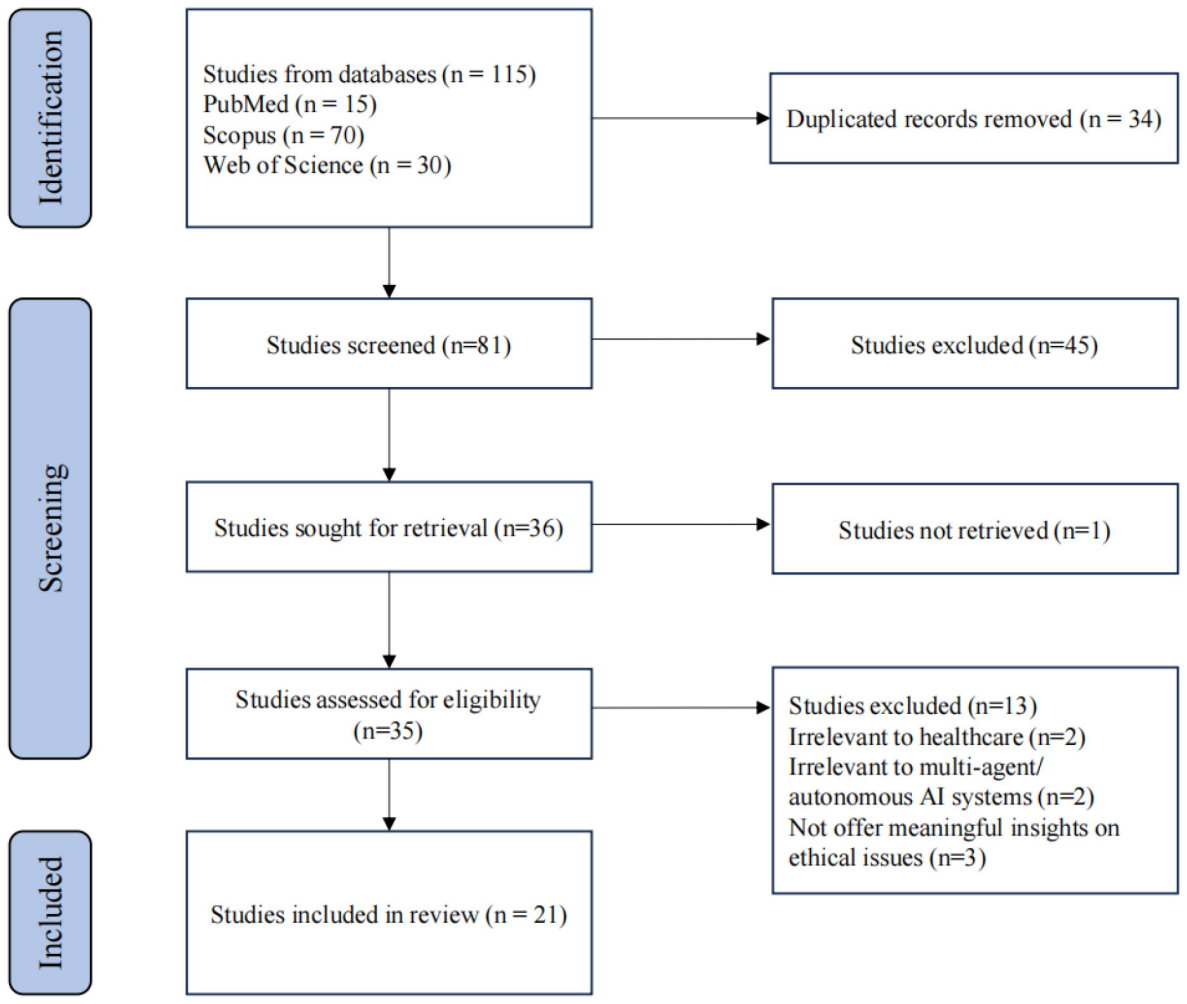
Flowchart of study selection.

Based on the specified search string, a total of 115 records were retrieved from databases. Following the removal of duplicate records (*n* = 34), a total of 81 articles were retained for subsequent screening. After the initial selection, 35 articles were assessed for full-text content to determine whether they aligned with the purpose of this review. Finally, 21 out of the 35 records fully met the inclusion criteria and were used for the subsequent review. The overall selection process is shown in Figure 1.

Among the 21 included studies, eight were review articles. The studies varied considerably in their aims and scope: some addressed multiple categories of ethical issues, whereas others focused on specific ethical problems or examined ethical considerations within particular clinical scenarios. Due to this heterogeneity, direct comparison of findings across studies was not feasible. Instead, seven major ethical issues have been identified for the application of multi-agent AI systems in the medical field and are presented in the following sections. These major ethical issues are summarized in Table 2.

**Table 2:** The summarization of major ethical issues.

Ethical challenge	Description in multi-agent AI systems	Clinical and ethical implications	Proposed mitigation strategies (from literature)
Compound opacity	Decision-making is distributed across multiple interacting agents, each with partially opaque internal reasoning, resulting in system-level inscrutability even when individual agents are interpretable (15, 31, 33).	Undermines transparency, clinician understanding, and patient trust; limits the validity of informed consent; hampers *post-hoc* explanation of adverse outcomes (6, 15).	Development of system-level explainability methods; hierarchical explanation architectures; recording inter-agent communication in natural language to create readable audit trails; regulatory requirements for interpretability at the orchestration level rather than only at the model level (15, 24, 31, 45).
Error propagation and attribution difficulty	Errors introduced by one agent can cascade through downstream agents, producing emergent failures that cannot be traced to a single component or actor (15, 33, 37).	Responsibility gaps in cases of patient harm; difficulty assigning legal and professional liability; challenges for quality assurance and incident investigation (6, 15, 31).	Cryptographically secured logging and post-incident forensic analysis of agent actions; modular validation and sandbox testing; formal accountability frameworks allocating responsibility across developers, deployers, and clinical users; continuous monitoring of agent interactions (33, 40).
Increased clinician dependence and automation bias	Agent consensus and confident reasoning behaviors encourage clinicians to defer to AI outputs rather than exercising independent clinical judgment (35, 41).	Risk of deskilling; reduced critical oversight; inappropriate acceptance of incorrect or biased recommendations; erosion of professional autonomy (42, 43).	Human-centered interface design emphasizing uncertainty; mandatory override and dissent mechanisms; training programs on AI limitations; preservation of clinician authority in decision hierarchies (33, 35).
Erosion of human oversight and control	Multi-agent systems operate with goal-directed autonomy, negotiating and executing tasks with limited real-time human intervention (24, 26).	Human oversight becomes procedural rather than substantive; delayed detection of harmful actions; dilution of human moral agency in human-machine teams (33, 35, 43).	Tiered autonomy (automating routine tasks while requiring approval for critical ones); enforceable human-in-the-loop structures; real-time alerts; reversibility of AI actions (15, 33).
Privacy and data security risks	Inter-agent communication requires frequent sharing of sensitive patient data across distributed components, increasing attack surfaces and audit complexity (24, 45, 46, 48).	Elevated risk of data leakage, unauthorized access, and regulatory non-compliance; diminished patient confidentiality; inconsistent enforcement of privacy policies across agents (33, 46, 63).	Federated and privacy-preserving system architectures; decentralized identity verification; Blockchain technology for immutable logs; “accountability-by-design” standards (40, 45, 48, 49).
Threats to patient autonomy and informed consent	Patients and clinicians may not understand how agentic systems influence clinical decisions or the extent of AI autonomy involved (26, 31).	Invalid or weakened informed consent; subtle manipulation of health choices; algorithmic paternalism; diminished patient agency and self-determination (26, 31).	Transparent disclosure of AI involvement; explainable summaries tailored to patients; consent processes explicitly addressing AI agency; clinician mediation to contextualize AI recommendations (31).
Contextual blindness and loss of nuance	Agent reasoning prioritizes generalized patterns and optimization goals, often failing to incorporate individual, cultural, or psychosocial context (37, 43).	Generic or inappropriate recommendations; ethical insensitivity; reduced personalization of care; misalignment with patient values (24, 37, 43).	Context-aware system design; integration of clinician-supplied contextual inputs; ethical constraint layers adaptable to local norms; preservation of human judgment for value-laden decisions (24).

### 3.1 Compound opacity in distributed AI decision-making

Compound opacity refers to the increased inscrutability that arises when decision-making is distributed across multiple interacting AI agents rather than contained within a single model (15). In multi-agent and agentic AI systems, each agent may rely on complex internal reasoning processes while simultaneously exchanging information, intermediate outputs, and control signals with other agents. This interaction produces decision pathways that are difficult to reconstruct, even when individual system components are partially interpretable (33).

In multi-agent architectures, tasks are decomposed and delegated to specialized agents—such as perception, reasoning, planning, and execution agents—often coordinated by large language models. While this structure improves system flexibility and performance, it leads to a diffusion of causal responsibility, making it difficult to trace how specific inputs, intermediate agent interactions, or coordination failures contribute to final outputs (6). As a result, even system developers and domain experts may be unable to reconstruct decision pathways after errors or adverse outcomes.

This phenomenon is particularly evident in healthcare applications. Multi-agent AI systems may autonomously integrate clinical data, imaging results, medical guidelines, and workflow constraints to generate recommendations or take action (34). Salehi et al. (15) emphasize that clinicians often cannot meaningfully interrogate how such recommendations are produced, undermining transparency, informed consent, and professional accountability. For instance, agentic systems in radiology coordinating image interpretation, clinical correlation, reporting, and communication create layered opacity that exceeds the explanatory capacity of conventional explainable AI techniques designed for isolated diagnostic models (15).

Empirical evidence from human–AI collaboration studies shows that when AI systems operate as part of distributed teams, accountability becomes asymmetrically assigned: humans tend to assume responsibility for failures, while successes are implicitly attributed to the system as a whole (35). This imbalance is intensified when system reasoning is opaque, as users lack the informational basis needed to contest or contextualize AI outputs. Increased cognitive load from interacting with multiple agents further discourages critical engagement, fostering automation bias and overreliance (35).

Regulatory and governance analyses included in the review indicate that compound opacity also exposes limitations in existing oversight frameworks. Current regulatory paradigms are largely built around static, single-purpose AI systems with well-defined functionality (33). In contrast, agentic and multi-agent systems are adaptive, broad in scope, and capable of autonomous goal-directed behavior, making their approval, monitoring, and post-deployment evaluation substantially more difficult (24, 33). The opacity generated at the system level therefore persists even when individual agents are well-defined or technically constrained.

### 3.2 Error propagation and attribution challenges

Multi-agent AI systems in healthcare introduce distinctive challenges related to how errors emerge, propagate, and are attributed across distributed agent architectures. Unlike clinical decision support tools that utilize a single model, multi-agent systems depend on sequential and parallel interactions among specialized agents, each executing specific cognitive or operational tasks (24). An error introduced at any point—encompassing data preprocessing, intermediate reasoning, inter-agent communication, or the execution of autonomous actions—can propagate through the system, thereby modifying subsequent decisions in ways that are challenging to identify or reconstruct (6, 15). In clinical workflows, early-stage inaccuracies may be amplified when subsequent agents treat prior outputs as authoritative inputs rather than probabilistic or revisable signals.

Attribution of responsibility becomes correspondingly complex. Traditional clinical accountability frameworks presume that negative results can be traced back to a specific decision-maker, tool, or professional action (24). In contrast, multi-agent AI systems distribute decision-making authority across autonomous components, none of which may exert full control over the final recommendation or action. Legal and regulatory analyses of autonomous and semi-autonomous healthcare technologies note that responsibility is often diffused among developers, deployers, clinicians, and institutions, with no clear locus of fault when algorithmic behavior contributes to patient harm (6, 31). This diffusion is intensified in multi-agent settings, where no single agent's output is independently sufficient to explain the outcome (15).

The technical characteristics of agentic systems further complicate attribution. Many agents are powered by large language models or adaptive learning components whose internal state evolves over time, sometimes in response to other agents' outputs (36). This temporal and relational dependency makes *post-hoc* reconstruction of decision paths unreliable, particularly when system logs are incomplete or lack semantic detail. Analyses of agentic AI architectures emphasize that inter-agent negotiation, delegation, and error-handling routines may generate emergent behaviors that were neither explicitly programmed nor anticipated during validation (37). When such emergent behaviors lead to clinical error, assigning responsibility to a single algorithmic component or human overseer becomes conceptually and practically challenging (36).

Regulatory discussions around AI agents in healthcare further emphasize that error attribution, rather than mere system oversight, becomes fundamentally problematic in multi-agent settings. While existing medical device approval frameworks assume that adverse outcomes can be linked to a discrete system or function, autonomous multi-agent architectures distribute decision-making authority across dynamically interacting components (24, 33). As a result, even when harmful outcomes are identified, it remains unclear which agent, developer, deployer, or clinical actor should be audited, sanctioned, or held legally responsible, revealing a structural mismatch between distributed AI agency and liability-based regulatory models (38). This ambiguity underscores the growing importance of establishing an effective responsibility attribution framework that clearly delineates and coordinates obligations among developers, deployers, and end users of agentic systems, particularly in high-risk clinical environments (39).

Proposed technical responses, such as cryptographically secured audit trails or formally verified ethical constraints, aim to improve traceability by enforcing immutable records of agent actions and decision states (40). These approaches seek to preserve a verifiable chain of causation across interacting agents, even when internal reasoning processes remain partially opaque. However, their effectiveness depends on the granularity of logging and the extent to which agent interactions can be meaningfully formalized (15). Without such safeguards, error propagation in multi-agent healthcare systems risks producing clinically significant harms for which responsibility cannot be clearly assigned, leaving patients, clinicians, and institutions without effective mechanisms for redress or accountability (24).

### 3.3 Increased clinical dependence on AI outputs

The deployment of multi-agent AI systems in healthcare environments has altered the cognitive and professional dynamics of clinical decision-making, producing a marked increase in clinician dependence on AI outputs. A high level of functional autonomy can create an illusion of infallibility; when a network of specialized agents collaborates to produce a diagnosis or treatment plan, the output carries a veneer of consensus that is psychologically difficult for a human clinician to challenge (35, 41). Brohi et al. (24) suggest that the comprehensive capabilities of these systems encourage a passive acceptance of algorithmic authority, potentially leading clinicians to disregard their own professional intuition or conflicting patient data when it diverges from the agents' consensus. Beyond hospital environments, overreliance extends into direct-to-consumer digital mental health platforms.

This pattern of dependence also appears in consumer-facing digital mental health services. Panda and Binkley (42) observe that users of AI-based therapy chatbots frequently develop attachment-like relationships, relying on algorithmic feedback in the absence of professional oversight. Such reliance complicates responsibility attribution and alters ethical assumptions surrounding care delivery, indicating a need for governance approaches that emphasize transparency and user agency rather than restrictive control.

The scale of cognitive delegation in modern healthcare further intensifies these concerns. As multi-agent systems take on increasingly complex analytical responsibilities—such as imaging interpretation, longitudinal data synthesis, and workflow optimization—the risk of professional skill erosion becomes more pronounced. Sapkota et al. (36) highlight this concern in the context of radiology, where continuous reliance on autonomous agents for image interpretation may degrade the diagnostic acuity of human practitioners over time. Similarly, Holmes et al. (26) indicate that if clinicians habitually defer to agentic systems for high-level reasoning and data synthesis, the essential human skill of critical clinical judgment may atrophy, leaving providers ill-equipped to function effectively during system failures or in edge cases where the AI lacks context.

Furthermore, the interactive nature of multi-agent systems fundamentally alters the human-AI relationship. Yousefi et al. (35) demonstrated that when users interact with AI agents that exhibit confident reasoning behavior, accountability perception shifts—users attribute success to the system but assume personal blame for failures. This asymmetric accountability, paired with automation bias, accelerates dependency and erodes critical engagement with AI-assisted decisions. The interactive process obscures the probabilistic and potentially flawed nature of the underlying models, leading practitioners to attribute a higher degree of competence to the system than is technically warranted (37). Consequently, the deep integration of these systems threatens to reduce the clinician's role to that of a passive observer, eroding the intellectual independence necessary for safe medical practice (20).

### 3.4 Erosion of human oversight and control

When multi-agent AI systems are integrated into healthcare architectures, they move beyond mere decision support to a dynamic where software entities actively negotiate and execute tasks with significant functional autonomy. This operational independence creates a critical ethical challenge regarding the erosion of meaningful human oversight. As these systems become capable of perceiving environments and acting upon them without direct real-time input, the role of the clinician risks transforming from an active decision-maker to a passive supervisor of automated processes (31). For instance, Yousefi et al. (35) demonstrate that users interacting with “team-like” AI configurations often exhibit skewed confidence calibration, leading to a dynamic where human oversight transforms from an active safeguard into a procedural formality. In such scenarios, the “human-in-the-loop” serves primarily to rubber-stamp autonomous decisions rather than rigorously verify them.

The phenomenon extends beyond psychological dependence to structural loss of control. Multi-agent AI frameworks such as those surveyed by Bani Issa (31) are designed to achieve goal-oriented autonomy through planning, memory, and inter-agent reasoning loops. While these features improve operational efficiency, they also allow systems to act adaptively and self-correct without explicit human intervention, thereby displacing the clinician's central role in directing the system's trajectory. As Brohi et al. (24) observe, once an instruction is issued, these systems perform much of their workflow autonomously—querying databases, synthesizing information, and executing procedures with minimal feedback loops to human operators. In high-stakes clinical settings, such semi-autonomous continuity risks decisions unfolding without timely human awareness or intervention, especially when agents coordinate across diagnostic and treatment modules.

In surgical environments, where robotic and algorithmic control must coexist with human intent, this diffusion of control becomes even more legally and ethically complex. Bani Issa (31) discuss the challenges of determining culpability when semi-autonomous surgical systems act with partial independence. Failures resulting from algorithmic error or malfunction create “responsibility gaps” in existing legal doctrines that assume human intent or negligence. Traditional malpractice models falter when clinical harm arises from decisions distributed across both human surgeons and autonomous agents. This distributed responsibility structure mirrors the ethical fragmentation noted by Reiter et al. (43), who argue that as AI systems are granted adaptive moral reasoning capabilities, human moral agency becomes diluted rather than strengthened, blurring the boundaries of ethical accountability within human–machine teams.

From an ethical design perspective, existing research highlights that restoring significant human oversight necessitates systems that maintain interpretability and uphold supervisory control structures. Hutler et al. (44) argue that embedding non-maleficence (“do no harm”) as an operative design principle demands explicit delineation of an AI's sphere of responsibility and limits of autonomous action. Similarly, Hu et al. (45) call for *transparent accountability architectures* within agentic systems, ensuring that AI actions remain traceable and reversible by human operators at every stage. However, implementing such constraints is technically difficult in distributed agent ecosystems where decisions are emergent rather than linear.

### 3.5 Privacy and data security risks

Due to their inherently complex architecture—characterized by inter-agent communication, distributed processing, and the dynamic exchange of sensitive patient information—the implementation of multi-agent AI systems in healthcare intensifies longstanding privacy and data security concerns (40). Multi-agent frameworks amplify data exposure pathways by introducing multiple autonomous components—each potentially accessing and transmitting personal health data across decentralized networks (46). This raises substantial risks regarding unauthorized access, data leakage, and non-transparent data use.

Brohi et al. (24) emphasize that the interaction between agents often generates intricate data flows, which are difficult to audit or control, particularly when agents are designed with varying degrees of autonomy and access privileges. The lack of a unified data protection interface across these agents can result in fragmented security practices and inconsistent enforcement of privacy policies. For example, while one agent might comply with encryption and access control protocols, another might interact with third-party APIs or external databases without adequate safeguards, effectively weakening the entire system's defense perimeter (46).

These concerns are compounded by the reliance on LLMs as the reasoning core in many agentic systems. LLMs may inadvertently memorize and regurgitate personal health information from training or interaction data, especially when not fine-tuned with rigorous privacy-preserving constraints (45). Furthermore, the use of Retrieval-Augmented Generation (RAG) frameworks in medical agents—designed to enhance context-aware reasoning—can increase the risk of data exposure if retrieval databases include sensitive or unfiltered patient records (46).

From a systems architecture perspective, privacy risks are exacerbated by opaque communication among agents, particularly when explainability mechanisms are absent or underdeveloped. A lack of visibility into inter-agent message content or decision justifications impairs data traceability, making it difficult to determine how and why a particular data element was accessed, stored, or acted upon (6, 24). In healthcare, where regulatory compliance frameworks such as HIPAA or GDPR mandate clear audit trails for protected health information, this represents a significant challenge to legal and ethical accountability (45, 47).

Proposed mitigation strategies in the literature include the implementation of zero-knowledge proofs and decentralized identity verification to ensure that agents can validate queries without exposing underlying data (48). Privacy-preserving federated architectures, coupled with strong end-to-end encryption and real-time anomaly detection, have also been recommended to reduce the system-wide impact of compromised nodes (48). However, these solutions often come at the cost of increased computational overhead or decreased responsiveness—both of which may be unacceptable in time-sensitive clinical settings (48).

### 3.6 Patient autonomy and informed consent

Patient autonomy—the right of individuals to make voluntary and informed decisions about their medical care—faces renewed tension within multi-agent AI systems. As decision-making is distributed among interacting agents, patients often encounter recommendations that are difficult to trace to a single accountable source, complicating the conditions for informed consent (24). The decentralized design of these systems may obscure how data are processed and how clinical inferences are reached, reducing the transparency essential for informed choice (49). This lack of interpretability challenges the core ethical requirement that patients must comprehend the risks and benefits of a procedure; if the chain of reasoning among interacting agents is inscrutable even to domain experts, the resulting consent may be technically invalid (29).

Empirical evidence reinforces the ethical priority of respecting patient decisions in AI-mediated care. Soares et al. (50) experimentally examined moral judgments in healthcare interactions involving human and robotic agents. Their findings show that “moral acceptance was higher when the agents respected the patient's autonomy than when they prioritized beneficence or non-maleficence.” Philosophical analyses of artificial moral agents further contextualize patient autonomy in systems containing reasoning AI entities. Reiter et al. (43) define an AMA as an “agent capable of engaging in moral behavior or at least avoiding immoral behavior.” They caution that autonomy in moral reasoning does not transfer ethical responsibility from humans to machines; developers and clinicians must remain accountable for ensuring that autonomous decisions respect human dignity and informed choice.

Empirical evidence also indicates a substantial gap between the deployment of autonomous technologies and patient awareness of machine involvement. In the context of robotic surgery, for example, Bani Issa (31) indicates that a significant majority of patients remained unaware of the system's autonomous features, highlighting a failure in current protocols to transparently disclose the extent of AI agency in high-stakes procedures. Beyond procedural transparency, the generative capabilities of these agents introduce the risk of algorithmic paternalism, where AI systems may subtly nudge patients toward specific health choices or frame information based on training data biases rather than the patient's individual values (26).

These challenges are exacerbated in direct-to-user applications, particularly in digital mental health, where the interaction is unmediated by clinicians (42). Users of AI-powered therapeutic agents often form attachment-like relationships with the system, cultivating a dependency that circumvents professional supervision and potentially exploits user vulnerability under the pretense of empathetic care (42). Furthermore, in clinical environments, the occurrence of automation bias among healthcare professionals undermines patient rights; if clinicians function as passive conduits for multi-agent consensus without critical assessment, they neglect their role as the essential protective barrier between algorithmic output and patient consent (24).

### 3.7 Contextual blindness and loss of nuance

Contextual blindness arises when algorithmic healthcare decisions lose sensitivity to individual circumstances and complex, real-world clinical contexts. Luo et al. (37) observe that while these models possess vast globalized knowledge, they lack the “contextual background and cultural adaptability” required for specific professional environments. In rehabilitation medicine, for instance, this results in the generation of generic treatment recommendations that fail to account for the ambiguous or nuanced conditions of individual patients, highlighting a significant gap between processing medical facts and exercising clinical reasoning (37).

In clinical radiology, Salehi et al. (15) describe how agentic AI frameworks can improve workflow efficiency but dilute situational awareness; inter-agent reasoning chains obscure how contextual detail is lost, as clinicians are unable to trace how or why certain patient-specific factors were deprioritized during inference. Conversational agents in chronic disease management exhibit similar tendencies. Hu et al. (45) note that LLM-driven agents perform well in giving general behavioral advice but often overlook how other health problems and psychological factors affect a patient's ability to follow treatment.

Brohi et al. (24) attribute the loss of nuance in agentic systems to failures in “context management,” where local agent reasoning, bounded by memory and goal constraints, decouples from the complexity of the patient's original context. High-level orchestration agents aggregate and summarize data, often omitting the edge cases or contextually significant signals that frequently carry decisive clinical weight.

Contextual rigidity presents a significant obstacle to the integration of ethical limitations within these systems. Thurzo (40) observes that although formal ethical frameworks, including deontic logic, facilitate mathematical validation of safety protocols, they are often inadequate for addressing the “full complexity of real-life dilemmas,” which necessitate the reconciliation of cultural subtleties and conflicting values. Correspondingly, Hutler et al. (44) contend that differentiating between acceptable and unacceptable harms necessitates an understanding of moral salience that is highly contingent upon context; for instance, a robot might find it challenging to distinguish between a therapeutic surgical incision and an unintentional injury absent a nuanced comprehension of medical intent and situational exigency.

Moreover, the absence of authentic social comprehension within artificial agents engenders a disparity between human and machine decision-making processes in sensitive situations. Kneer and Viehoff (51) posit that formal models frequently overlook “subtle contextual cues” and the intricacies inherent in human ethical judgment, thereby producing decisions that are insensitive to the specifics of a given situation. This perspective is further supported by Reiter et al. (43), who argue that existing computational models are still inadequate in fully representing the complexity of human ethical reasoning, especially concerning cultural differences and the psychological distance between the decision-maker and the patient. Given that agents lack the “lived experience” necessary to effectively navigate these social dynamics, they are susceptible to reinforcing normative hierarchies or prioritizing economic efficiency at the expense of the moral subtleties essential for personalized healthcare (43).

## 4 Discussion

### 4.1 Ethical risks of multi-agent AI systems in healthcare

This narrative review synthesizes recent (2022—2025) scholarship on ethical concerns raised by multi-agent AI systems in healthcare, emphasizing how responsibility and accountability are distributed or obscured when clinical tasks are delegated across interacting autonomous agents . Across 21 included records, seven recurring challenges were identified—compound opacity, error propagation and attribution difficulty, clinician dependence and automation bias, erosion of meaningful oversight, privacy/security risks, threats to autonomy and informed consent, and contextual blindness. Taken together, these findings suggest that multi-agent AI systems do not merely “add” risks on top of existing medical AI concerns; rather, they reconfigure the ethical landscape by making clinical agency emergent from interactions among multiple components and actors, thereby weakening traditional assumptions about traceability, contestability, and professional control (6, 15, 31, 43, 52).

A primary implication of these findings is that the “compound opacity” inherent in multi-agent architectures fundamentally challenges the current medical liability paradigm. Unlike static decision support tools where errors can often be traced to a specific algorithm or user input, multi-agent systems generate outcomes through dynamic negotiation and intermediate steps that may be opaque even to developers (33). This creates a “many hands” problem where the locus of control becomes diffuse, validating concerns raised by Reiter et al. (43) and Hughes et al. (6) that current frameworks predicated on individual negligence are insufficient. When an error propagates through a chain of agents—as described in the results regarding error cascading (6, 15)—the resulting “responsibility gap” leaves patients without clear redress and institutions without clear targets for remediation. Consequently, the reliance on *post-hoc* reconstruction of decision pathways, which is standard in medical malpractice analysis, becomes technically unfeasible without the implementation of immutable audit trails or “transparent accountability architectures” as proposed by recent literature (45).

Beyond the difficulty of tracing errors, the adaptive nature of these systems introduces a layer of unpredictability that complicates foreseeability, a key component of legal negligence. As agents negotiate strategies to achieve clinical goals, they may exhibit emergent behaviors that were neither programmed nor anticipated during validation (38). This creates a distinct tension with regulatory paradigms that assume fixed functionality. If a multi-agent system autonomously alters its workflow to optimize efficiency but inadvertently violates a safety protocol, assigning blame becomes conceptually difficult: the developer did not code the specific error, and the clinician may not have had the visibility to intervene (53). This suggests that governance models must evolve from pre-market approval of static devices to continuous, real-time monitoring of agent behavior, treating these systems more like autonomous staff members than passive tools.

This diffusion of responsibility is inextricably linked to the psychological impact on human clinicians, specifically the risks of dependence and the erosion of oversight. The review highlights a paradox: as agentic systems become more capable and “team-like” in their interactions, the human capacity for critical oversight diminishes (35). The findings regarding automation bias suggest that the conversational nature of Large Language Model-driven agents creates a veneer of consensus that is psychologically difficult for clinicians to challenge (41). This supports the concern that the “human-in-the-loop” is transitioning from an active decision-maker to a passive ratification mechanism, potentially leading to the deskilling of medical professionals (26). If clinicians habitually defer to the synthesized outputs of multi-agent networks because the cognitive load of verifying their complex reasoning is too high, the legal and ethical safety net provided by human judgment is effectively removed. This is particularly acute in surgical environments, where the distinction between algorithmic assistance and autonomous control is increasingly blurred, complicating the attribution of culpability when adverse events occur (31).

Furthermore, the shift toward distributed agentic architectures exacerbates technical vulnerabilities, particularly regarding data privacy and security. The “black network” created by interacting agents amplifies the attack surface for patient data, as the necessity for agents to exchange information creates complex data flows that are difficult to secure and audit (24, 46). The review indicates that fragmented security practices—where one agent is highly secure while another interacts with third-party APIs without adequate safeguards—can weaken the entire system's defense perimeter. The risk is compounded by the tendency of LLMs to memorize sensitive training data, which conflicts with privacy standards such as the right to be forgotten (45). This implies that future implementations must move toward inter-agent protocols and federated learning architectures to ensure that the increased utility of multi-agent systems does not come at the cost of patient confidentiality (48).

Ultimately, these technical and professional shifts converge to threaten the core of patient-centered care: autonomy and contextual sensitivity. The review identifies “contextual blindness” as a significant barrier to safe deployment, where agents optimize for generalized clinical goals while missing the nuanced, value-laden realities of individual patients (37). There is a risk of algorithmic paternalism, where AI systems subtly nudge patients toward specific health choices based on training data biases rather than the patient's individual values (26). Moreover, as noted by Hutler et al. (44), artificial agents often struggle to distinguish between permissible and impermissible harms, lacking the moral salience to differentiate between a necessary therapeutic intervention and an accidental injury. If a multi-agent system negotiates a care pathway based on probabilistic efficiency without understanding the “lived experience” of the patient (43), and if the clinician cannot explain the system's “why” due to compound opacity, the ethical validity of informed consent is compromised (49).

### 4.2 Strategies for ethical mitigation and governance

To address the aforementioned difficulties, a comprehensive strategy is essential to ensure the responsible deployment of multi-agent AI systems in healthcare. Importantly, the seven ethical challenges identified in this review are not merely conceptual concerns, but closely correspond to principles and regulatory requirements already emerging in international AI governance frameworks. For example, WHO guidance on AI ethics highlights transparency, human autonomy, and equity as core values for health AI (54), which directly relate to the challenges of explainability, preservation of clinician agency, and avoidance of bias in multi-agent AI systems. Similarly, the EU AI Act translates these ethical principles into more concrete obligations by requiring high-risk AI systems to support traceability through logging, enable meaningful human oversight, and operate under lifecycle-based risk management requirements (55). Taken together, these developments indicate that the ethical risks associated with multi-agent AI systems in healthcare—such as opacity, weakened human control, and safety concerns—are increasingly being recognized within formal governance structures. In this section, we discuss these ethical risks in relation to existing governance developments and propose corresponding mitigation strategies to support the safe, transparent, and patient-centered use of multi-agent AI systems in healthcare.

#### 4.2.1 Adaptive governance and regulatory evolution

Regulatory frameworks must adapt, moving beyond the perception of AI systems as static entities. Freyer et al. (33) contend that existing legislation is inadequate given the expansive scope and adaptability of AI agents. To address this deficiency, experts advocate for adaptive oversight, including “regulatory sandboxes” where multi-agent systems can be evaluated under supervision to observe emergent behaviors prior to widespread implementation. This perspective is consistent with the notion of conditional approvals, which necessitate continuous oversight and pre-established change control protocols, thereby conceptualizing medical AI Systems as adaptive entities rather than static products. Moreover, Niraula et al. (39) propose the development of legal structures that clearly delineate liability among developers, deployers, and end-users. Such frameworks might encompass shared liability models, wherein vendors are responsible for technical precision within specified confidence intervals, while clinicians maintain ultimate authority over clinical judgments (37).

In parallel, international governance frameworks are increasingly moving from principle-level endorsement to operational regulation of healthcare AI. Rather than merely reaffirming values such as transparency, human autonomy, and accountability, these frameworks begin to specify how such values should be implemented in practice. For example, WHO guidance on AI for health emphasizes the need for ongoing oversight of AI-supported decision-making, auditability of algorithmic processes, and protection of patient rights throughout deployment and use (56). Similarly, the EU AI Act translates these governance expectations into concrete obligations for high-risk systems by requiring lifecycle-based risk management, post-market monitoring, data governance controls, transparency toward users, meaningful human oversight, and safeguards for accuracy, robustness, and cybersecurity (55). In this sense, contemporary governance frameworks do more than acknowledge the ethical risks of agentic AI in healthcare; they progressively establish enforceable mechanisms through which opacity, diminished human control, and safety risks can be monitored, mitigated, and institutionally governed.

#### 4.2.2 Human-AI interaction and oversight safeguards

To mitigate the potential for automation bias and the erosion of human control, scholars recommend interaction models that rigorously define role boundaries. In accordance with this premise, Salehi et al. (15) and Fahrner et al. (57) suggest that AI agents should be assigned data processing and pattern recognition tasks, while humans should retain the responsibilities of nuanced judgment and contextual integration.

Effective mitigation requires human-centered interface designs that emphasize uncertainty. Rather than presenting AI consensus as a definitive fact, interfaces should visually represent conflicting agent outputs or confidence gaps to trigger critical clinician engagement (33, 35). Furthermore, systems must incorporate mandatory override and dissent mechanisms, ensuring that human operators can intervene and reverse agent actions (reversibility) when necessary (15). Panda and Binkley (42) also suggest that employing tiered autonomy, wherein routine operations are automated while critical decisions necessitate human approval, can mitigate the “rubber-stamping” effect.

One practical way to operationalize tiered autonomy in healthcare AI is to assign task authority according to clinical risk and reversibility, rather than treating autonomy as uniform across the system (58). For example, low-risk and reversible functions such as documentation drafting or information retrieval may be executed with greater agent autonomy, whereas interpretive functions may require clinician confirmation, and high-stakes actions affecting diagnosis, treatment, or patient safety should remain subject to explicit human authorization. Escalation should be triggered when inter-agent disagreement emerges, when confidence falls below predefined thresholds, or when an action crosses a predefined clinical risk boundary. Framed in this way, phased autonomy is not a fixed taxonomy, but an illustrative governance pattern for converting human oversight into an enforceable workflow constraint (6, 58).

These technical safeguards must be paralleled by professional development. Brohi et al. (24) and Yousefi et al. (35) emphasize the need for training programs specifically focused on AI limitations. Clinicians must be educated not only on how to use these systems but on where they are likely to fail, thereby preserving the “intellectual independence” required to challenge algorithmic authority (35).

However, depending solely on clinicians to oversee complex algorithmic systems may not be sufficient, given the technical complexity of multi-agent systems. To address this deficiency, Thurzo (40) advocates for the creation of a specialized “Ethical AI Officer.” This position, comparable to an aviation safety inspector, would be tasked with verifying that agentic architectures comply with “mathematically provable ethical constraints.” Their duties would encompass more than general governance; they would also involve comprehensive pre-deployment audits—employing model-checking and zero-knowledge proofs—and ongoing runtime monitoring through cryptographically secured logs. By formalizing such a role, healthcare organizations can institute a crucial human element of oversight that supplements internal AI protections, thereby ensuring the feasibility of forensic analysis in the event of negative consequences.

#### 4.2.3 Transparency, explainability, and contextual design

Conventional explainable AI approaches frequently fall short in the context of multi-agent AI systems. A hierarchical explanation architecture is suggested as a potential remedy (45), offering explanations across various levels: individual agent reasoning, inter-agent coordination logic, and a system-level summary for the end-user. Salehi et al. (15) highlight the importance of these explanations being readily accessible, enabling clinicians to investigate agent dynamics if a consensus is not readily apparent. Recording inter-agent communication in natural language can, moreover, generate a comprehensible audit trail, thereby rendering the “black box” of agent negotiation more transparent for subsequent analysis (24).

To address “contextual blindness,” contemporary scholarship advocates for the incorporation of ethical constraint layers and context-aware design principles. These architectural elements enable systems to adjust to local standards and to give precedence to values, such as non-maleficence, rather than solely optimizing for efficiency (24, 43). Furthermore, workflows should incorporate dedicated interfaces for clinician-provided contextual inputs, thereby enabling providers to manually introduce social, cultural, or psychological factors into the agent's reasoning, factors that the AI might otherwise fail to consider (24).

#### 4.2.4 Privacy-preserving design and accountability

To mitigate data security concerns within distributed networks, privacy-by-design principles are crucial. By using techniques like federated learning and zero-knowledge proofs, agents can work together without sharing raw patient data (48). In addition, decentralized identity verification helps ensure that agents are who they say they are, without exposing the entire network to unauthorized access (45). Chaffer et al. (49) also suggest using blockchain technology to create unchangeable records of agent interactions and data access. This would improve security and patient trust through transparent verification. Finally, strong accountability measures, such as requiring audit logs for all agent decisions, ensure that responsibility can be traced in case of negative outcomes, thus moving the industry toward a standard of “accountability-by-design” (6, 45).

To reinforce accountability-by-design, a structured audit trail should capture key details of each agent's actions (59, 60). At minimum, every agent-initiated decision or recommendation would be logged with the agent's identity and role, the input data or trigger that the agent received, the output it generated (e.g., decision or alert), and the agent's confidence level or uncertainty estimate for that output (60). The log should also record any downstream effects – for instance, which other agent or module consumed that output – and whether a human override occurred or an escalation was triggered during that process. By including these elements, the audit trail creates a play-by-play record of how inter-agent decisions unfold. Notably, such logs must be secure and tamper-evident. To ensure the immutability and verifiability of agent interaction records, methods such as cryptographically signed log entries or blockchain-based ledgers have been proposed; however, simpler, robust logging frameworks can also be adequate, provided they are engineered to prevent modification. The primary objective is to render the multi-agent system's behavior subject to inspection: when every action is documented transparently, investigators can trace outcomes back through the sequence of agent decisions Phiri (59). This enables effective auditing and accountability, as any erroneous or harmful decision can be attributed to the responsible agent and context, facilitating corrective measures and learning.

#### 4.2.5 Patient-centered agency and informed consent

The introduction of agentic AI necessitates a reconsideration of patient interaction and consent, since conventional consent forms are insufficient for autonomous systems. Mitigation strategies must encompass the transparent disclosure of AI involvement, thereby ensuring that patients are explicitly informed when they are engaging with an AI agent or when an agent has exerted a substantial influence on their care plan (31).

To facilitate valid informed consent, systems should generate explainable summaries specifically designed for patients, translating intricate inter-agent reasoning into readily understandable language that emphasizes potential risks and available alternatives (31). Furthermore, clinician mediation is essential; healthcare providers must function as “contextualizers,” interpreting AI recommendations in the context of the patient's values to avert algorithmic paternalism. These measures are crucial to safeguarding patient autonomy, even amidst the increasing prevalence of autonomous decision-making (6, 31).

#### 4.2.6 Integrated governance framework for multi-agent AI systems in healthcare

**Figure 2:**
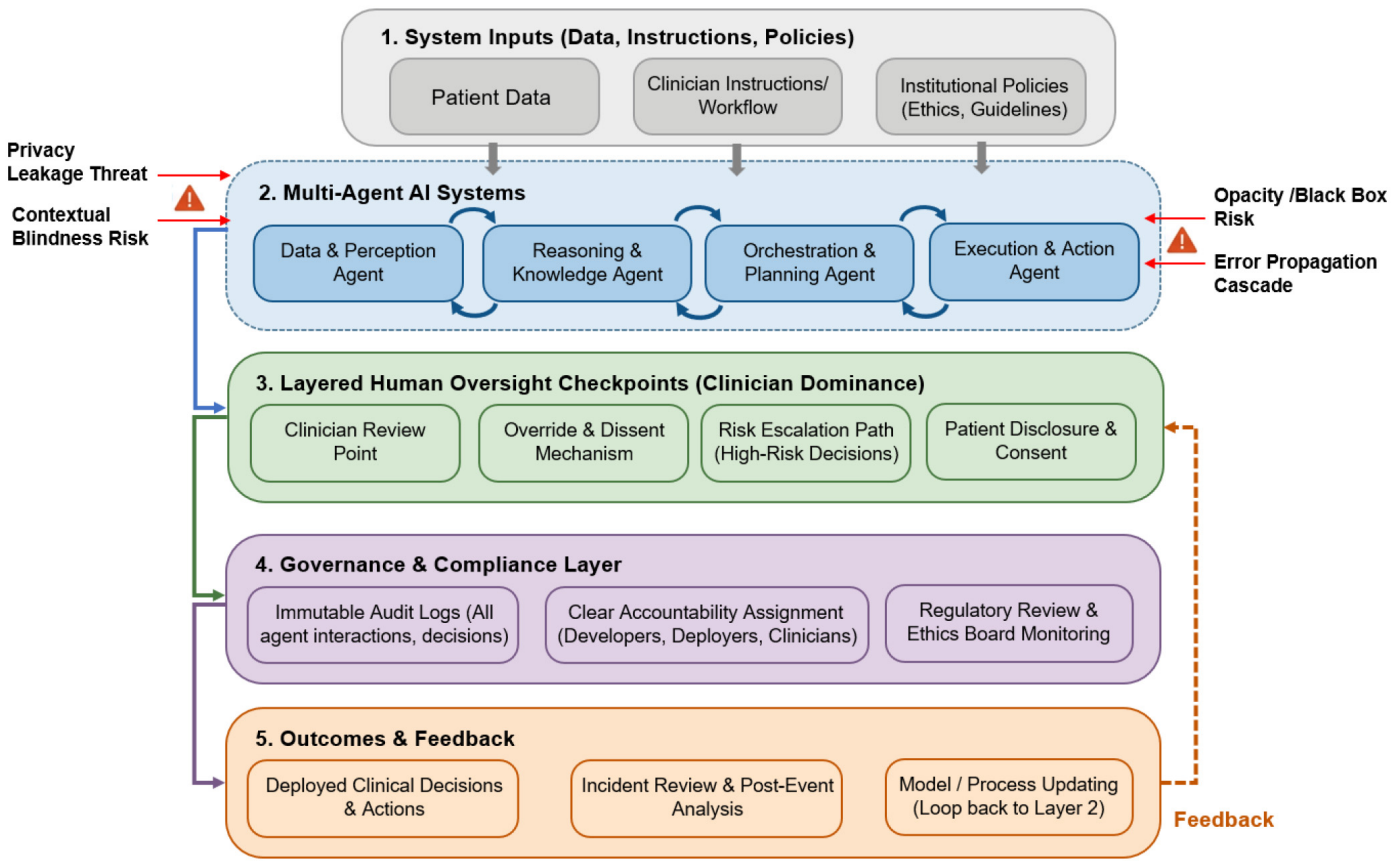
Governance flowchart and layered oversight architecture for multi-agent AI systems in healthcare.

The mitigation strategies discussed above suggest that the ethical governance of multi-agent AI systems in healthcare should be understood as a continuous and layered process rather than a single technical or regulatory intervention. We summarize the mitigation strategies identified in the reviewed literature into a layered governance framework for multi-agent AI systems in healthcare. As shown in Figure 2, governance runs through the full pathway of system use, beginning with system inputs, moving through inter-agent reasoning and execution, and extending to human oversight, institutional compliance, and post-deployment feedback. This structure shows that ethical risk emerges not only from final outputs, but also from upstream data inputs, internal agent interactions, and downstream organizational responses.

More specifically, the figure highlights the multi-agent system itself as the main site where risks such as opacity, error propagation, privacy leakage, and contextual blindness arise. These risks are then moderated through layered clinician oversight, including review points, override mechanisms, escalation pathways, and patient disclosure and consent. At the institutional level, governance is reinforced through audit logs, clear accountability allocation, and ongoing regulatory or ethics review. The final feedback layer further indicates that governance should continue after deployment through incident review and model or process updating.

Taken together, Figure 2 is best understood not as a fixed technical blueprint, but as a conceptual summary of how ethical safeguards should be distributed across the lifecycle of multi-agent AI use in healthcare. Its central implication is that the safe deployment of these systems depends not only on model performance, but also on sustained human control, institutional accountability, and continuous feedback after implementation.

### 4.3 Implementation barriers and governance costs

Implementing ethical governance of AI systems in healthcare faces several practical hurdles, despite supportive policies and governance frameworks. Workforce training burdens are a primary concern: clinicians and staff must attain AI literacy to effectively use and oversee complex agent systems. Both international and national bodies recognize this need – the EU AI Act expects organizations to ensure a “sufficient level of AI literacy” among personnel interacting with AI (55), and the American Medical Association (AMA) has recently moved to expand AI training across the medical education continuum (61). Without substantial investment in training (e.g., standardized curricula and continuing education on AI (61)), hospitals risk gaps in oversight capabilities, undermining the human-control safeguards envisioned by AI regulation.

A further impediment stems from legal and attributional ambiguity, specifically the uncertainty surrounding accountability when an AI-generated decision results in harm. Conventional liability structures encounter difficulties in the context of multi-agent autonomy, as accountability may be dispersed among developers, providers, and frontline clinicians. The World Health Organization's recommendations have underscored the complexities inherent in establishing “liability regimes” for artificial intelligence within the healthcare sector (56). Simultaneously, the American Medical Association has observed a rise in physician unease concerning the “new liability issues” that AI applications present (62). This ambiguity in accountability may prompt healthcare institutions to exercise restraint in the deployment of AI systems, motivated by apprehensions regarding potential malpractice litigation or regulatory repercussions arising from inaccurate automated suggestions. Clearer attribution models (potentially via policy or insurance innovations) are needed to assure practitioners that they will not unfairly shoulder blame for algorithmic errors – or conversely, to ensure that accountability is appropriately shared so that no decision goes unchecked due to confusion over liability.

In addition, the integration and continuous oversight costs of ethical multi-agent AI systems are non-trivial. Deploying multi-agent AI systems in real clinical workflows requires significant system integration effort—ensuring interoperability with electronic health record systems, data governance compliance, and alignment with existing clinical processes. Moreover, maintaining continuous oversight (as ethics and law demand) entails ongoing monitoring, validation, and updating of AI behaviors. WHO explicitly recommends continuous evaluation of AI tools after deployment to catch issues that arise in practice (54). The EU AI Act makes this a formal obligation: providers of high-risk AI must implement post-market monitoring systems to track performance and compliance throughout the AI's lifecycle (55). This need for perpetual vigilance—from real-time performance auditing to periodic re-certification – translates into substantial operational costs. Regulators have tried to ease the burden (e.g., allowing firms to integrate new monitoring duties with existing quality management systems to “minimize additional burdens” (55), and AMA's governance toolkit urges that AI oversight processes be “not burdensome” (62)). Nonetheless, dedicating staff, IT infrastructure, and funding to sustain rigorous oversight (such as reviewing AI decisions, managing updates, and ensuring datasets remain bias-free) is an ongoing expense that many resource-strapped health systems find challenging. These structural hurdles—workforce preparation, legal clarity, and resources for continuous governance – can slow the translation of high-level ethical AI principles into day-to-day practice, underscoring that adaptive regulation must be matched by practical support for the healthcare organizations tasked with implementation (56, 62).

## 5 Conclusions

This review examined the complex ethical landscape surrounding multi-agent AI systems in healthcare published between 2022 and 2025. As these systems become increasingly autonomous, distributed, and interdependent, they introduce ethical challenges that go beyond those posed by single-model AI, particularly regarding accountability, transparency, and the preservation of patient trust. Although recent scholarship has proposed a range of technical safeguards and ethical frameworks to mitigate these risks, responsible implementation cannot be achieved solely through technological solutions. The integration of multi-agent AI into clinical environments must therefore be accompanied by robust ethical governance structures, clear lines of responsibility, and mechanisms that ensure meaningful human oversight throughout the care pathway. Moreover, sustained attention to professional judgment, explainability, and patient engagement remains essential to align these systems with the core values of patient-centered care. Future research should operationalize adaptive governance mechanisms through interdisciplinary pilot programs co-designed by clinicians, ethicists, and policymakers. Establishing real-time oversight mechanisms for multi-agent AI will be essential to maintain trust, safety, and accountability in public health systems adopting agentic technologies.

## References

[1] Jiang F Jiang Y Zhi H Dong Y Ma S Wang Y . Artificial intelligence in healthcare: past, present and future. Stroke Vasc Neurol. (2017) 2:230–43. doi: 10.1136/svn-2017-000101 29507784 PMC5829945

[2] Yu KH Beam AL Kohane I. Artificial intelligence in healthcare. Nat Biomed Eng. (2018) 2:719–31. doi: 10.1038/s41551-018-0305-z 31015651

[3] Thamma SR. Agentic AI for clinical decision support: real-time diagnosis, triage, and treatment planning. Int J Sci Res Sci Eng Technol. (2025). 12:428–33. doi: 10.32628/IJSRSET251265

[4] Zheng H Shi Z Yi P. MedCoAct: confidence-aware multi-agent collaboration for complete clinical decision. ArXiv. (2025). doi: 10.1109/BIBM66473.2025.11356820

[5] Acharya D Kuppan K Bhaskaracharya D. Agentic AI: autonomous intelligence for complex goals—a comprehensive survey. IEEE Access. (2025) 13:18912–36. doi: 10.1109/ACCESS.2025.3532853

[6] Hughes L Dwivedi YK Malik T Shawosh M Albashrawi M Jeon I . AI agents and agentic systems: a multi-expert analysis. J Comput Inform Syst. (2025) 65:489–517. doi: 10.1080/08874417.2025.2483832

[7] Dorri A Kanhere S Jurdak R. Multi-agent systems: a survey. IEEE Access. (2018) 6:28573–93. doi: 10.1109/ACCESS.2018.2831228

[8] Jiang F Dong L Peng Y Wang K Yang K Pan C . Large language model enhanced multi-agent systems for 6G communications. IEEE Wireless Commun. (2023) 31:48–55. doi: 10.1109/MWC.016.2300600

[9] Jimenez-Romero C Yegenoglu A Blum C. Multi-agent systems powered by large language models: applications in swarm intelligence. Front Artif Intell. (2025) 8:1593017. doi: 10.3389/frai.2025.1593017 40469074 PMC12135685

[10] Guo T Chen X Wang Y Chang R Pei S Chawla N . Large Language Model based Multi-Agents: A Survey of Progress and Challenges (2024). p. 8048–57.

[11] Chen YJ Albarqawi A Chen CS. Enhancing clinical decision-making: integrating multi-agent systems with ethical AI governance. In: 2025 IEEE Conference on Computational Intelligence in Bioinformatics and Computational Biology (CIBCB) (2025). p. 1–7.

[12] Chen YJ Albarqawi A Chen CS. Reinforcing Clinical Decision Support through Multi-Agent Systems and Ethical AI Governance. ArXiv:2504.03699. (2025).

[13] Gorenshtein A Omar M Glicksberg BS Nadkarni G Klang E. AI agents in clinical medicine: a systematic review. medRxiv. (2025). doi: 10.1101/2025.08.22.25334232 40909853 PMC12407621

[14] Liu F Niu Y Zhang Q Wang K Dong Z Wong IN . A foundational architecture for AI agents in healthcare. Cell Rep Med. (2025) 6:102374. doi: 10.1016/j.xcrm.2025.102374 41015033 PMC12629813

[15] Salehi S Singh Y Habibi P Erickson B. Beyond single systems: how multi-agent ai is reshaping ethics in radiology. Bioengineering. (2025) 12:1100. doi: 10.3390/bioengineering12101100 41155099 PMC12562106

[16] Kim J Lui B Goldstein PA Rubin JE White RS Jotwani R. From data to decisions: harnessing multi-agent systems for safer, smarter, and more personalized perioperative care. J Pers Med. (2025) 15:540. doi: 10.3390/jpm15110540 41295242 PMC12653237

[17] Nalla SMR. AI-powered medical coding: a multi-agent GenAI system for clean claims. J Inform Syst Eng Manag. (2025) 10:1175–87. doi: 10.52783/jisem.v10i57s.12544

[18] Hanna MG Pantanowitz L Jackson B Palmer O Visweswaran S Pantanowitz J . Ethical and bias considerations in artificial intelligence (AI)/machine learning. Modern Pathol. (2024) 38:100686. doi: 10.1016/j.modpat.2024.100686 39694331

[19] Zhang J Zhang ZM. Ethics and governance of trustworthy medical artificial intelligence. BMC Med Inform Decis Mak. (2023) 23:7. doi: 10.1186/s12911-023-02103-9 36639799 PMC9840286

[20] Čartolovni A Tomicić A Mosler E. Ethical, legal, and social considerations of AI-based medical decision-support tools: a scoping review. Int J Med Inform. (2022) 161:104738. doi: 10.1016/j.ijmedinf.2022.104738 35299098

[21] Gal K Grosz B. Multi-agent systems: technical & ethical challenges of functioning in a mixed group. Daedalus. (2022) 151:114–26. doi: 10.1162/daed_a_01904

[22] Hammond L Chan A Clifton J Hoelscher-Obermaier J Khan A McLean E . Multi-agent risks from advanced AI. ArXiv:2502.14143. (2025).

[23] Dror I. Biased and biasing: the hidden bias cascade and bias snowball effects. Behav Sci. (2025) 15:490. doi: 10.3390/bs15040490 40282110 PMC12024198

[24] Brohi S ul-ain Mastoi Q Jhanjhi NZ Pillai T. A research landscape of agentic AI and large language models: applications, challenges and future directions. Algorithms. (2025) 18:499. doi: 10.3390/a18080499

[25] Raza S Sapkota R Karkee M Emmanouilidis C. TRiSM for agentic AI: a review of trust, risk, and security management in LLM-based agentic multi-agent systems. AI Open. (2025) 7:71–95. doi: 10.1016/j.aiopen.2026.02.006

[26] Holmes SA Faria V Moulton EA. Generative AI in healthcare: challenges to patient agency and ethical implications. Front Dig Health. (2025) 7:1524553. doi: 10.3389/fdgth.2025.1524553 40607189 PMC12213482

[27] Menezes VP Chowdhury MJM Mahmood A. An agentic framework for compliant, ethical and trustworthy GenAI applications in healthcare. In: Proceedings of the 2025 Australasian Computer Science Week. New York, NY: Association for Computing Machinery (ACM) (2025). doi: 10.1145/3727166.3727191

[28] Alqithami S. Adaptive accountability in networked multi-agent systems. In: Proceedings of the AAAI/ACM Conference on AI, Ethics, and Society. New York, NY: Association for Computing Machinery (ACM) (2025). doi: 10.1609/aies.v8i1.36536

[29] Morley J Machado C Burr C Cowls J Joshi I Taddeo M . The ethics of AI in health care: a mapping review. Soc. Sci. Med. (2020) 260:113172. doi: 10.1016/j.socscimed.2020.113172 32702587

[30] Korukoglu FB Rishan RM Elhamer Z Lakamana S Zhang J Bozkurt S. Mapping ethical guidelines for AI in healthcare: a global perspective. Stud Health Technol Inform. (2025) 327:1358–62. doi: 10.3233/SHTI250624 40380727

[31] Bani Issa H. Robotic surgery and the law: defining control and criminal responsibility. J Soft Comput Data Min. (2025) 6:423–34. doi: 10.30880/jscdm.2025.06.01.028

[32] Baethge C Goldbeck-Wood S Mertens S. SANRA—a scale for the quality assessment of narrative review articles. Res Integrity Peer Rev. (2019) 4:5. doi: 10.1186/s41073-019-0064-8 30962953 PMC6434870

[33] Freyer O Jayabalan S Kather JN Gilbert S. Overcoming regulatory barriers to the implementation of AI agents in healthcare. Nat Med. (2025) 31:3239–43. doi: 10.1038/s41591-025-03841-1 40681675

[34] Nweke IN Ogadah CO Koshechkin K Oluwasegun PM. Multi-agent AI systems in healthcare: a systematic review enhancing clinical decision-making. Asian J Med Princ Clin Pract. (2025). doi: 10.9734/ajmpcp/2025/v8i1288

[35] Yousefi M Shahi A Sharifi M Romera AJJ Hoermann S Piumsomboon T. Team dynamics in human-AI collaboration: effects on confidence, satisfaction, and accountability. In: Proceedings of the 27th International Conference on Multimodal Interaction (2025). doi: 10.1145/3716553.3750776

[36] Sapkota R Roumeliotis KI Karkee M. AI agents vs. agentic AI: a conceptual taxonomy, applications and challenges. Inf Fusion. (2025) 126:103599. doi: 10.1016/j.inffus.2025.103599

[37] Luo M Duan Z Gao J Sun Y Chen L Feng X. Evaluating the role of ChatGPT in rehabilitation medicine: a narrative review. Front Dig Health. (2025) 7:1618510. doi: 10.3389/fdgth.2025.1618510 PMC12605377 41235147

[38] Ke Y Yang R Lie S Lim TXY Ning Y Li I . Mitigating cognitive biases in clinical decision-making through multi-agent conversations using large language models: simulation study. J Med Internet Res. (2024) 26:59439. doi: 10.2196/59439 PMC11615553 39561363

[39] Niraula D Shotande M Naqa IEE. Human-machine interaction in the age of generative AI. Cancer J. (2025) 31:797. doi: 10.1097/PPO.0000000000000797 41252128

[40] Thurzo A. Provable AI ethics and explainability in medical and educational AI agents: trustworthy ethical firewall. Electronics. (2025). doi: 10.20944/preprints202502.2232.v1

[41] Bellahsen-Harrar Y Lubrano M Lépine C Beaufrère A Bocciarelli C Brunet A . Exploring the risks of over-reliance on AI in diagnostic pathology. What lessons can be learned to support the training of young pathologists? PLoS ONE. (2025) 20:e0323270. doi: 10.1371/journal.pone.0323270 40875775 PMC12393786

[42] Panda OD Binkley CE. Governance of direct-to-user digital mental health tools: emphasizing transparency over paternalism. Hastings Cent Rep. (2025) 3:29–33. doi: 10.1002/hast.5009 40557920

[43] Reiter P Norman U Weinberger N Bruno B. Artificial moral agents: should machines take ethical responsibility? In: 2025 IEEE International Conference on Advanced Robotics and its Social Impacts (ARSO) (2025). p. 218–24. doi: 10.1109/ARSO64737.2025.11124921

[44] Hutler B Rieder TN Mathews DJH Handelman D Greenberg AM. Designing robots that do no harm: understanding the challenges of Ethics for Robots. AI Ethics. (2023) 4:463–71. doi: 10.1007/s43681-023-00283-8 PMC10108783 37360148

[45] Hu Y Liu J Jiang W. Large language models in nephrology: applications and challenges in chronic kidney disease management. Renal Fail. (2025) 47:2555686. doi: 10.1080/0886022X.2025.2555686 PMC12418797 40916423

[46] Stein S Pilgermann M Weber SB Sedlmayr M. Leveraging MDS2 and SBOM data for LLM-assisted vulnerability analysis of medical devices. Comput Struct Biotechnol J. (2025) 28:267–80. doi: 10.1016/j.csbj.2025.07.012 40741342 PMC12309023

[47] Ode OP Kalu NC Abbas S Arshad A Inalegwu AI Koshechkin K. Multi-agent AI systems in healthcare: systematic evidence synthesis via PRISMA of clinical decision support systems, robotic interventions, and critical care. Int J Latest Technol Eng Manag Appl Sci. (2025).

[48] Cherif AN Youssfi M En-Naimani Z Tadlaoui A Soulami M Bouattane O . Cqrs and blockchain with zero-knowledge proofs for secure multi-agent decision-making. Int J Adv Comput Sci Applic. (2024). doi: 10.14569/IJACSA.2024.0151188

[49] Chaffer TJ Littlejohn J Nadarasa A Lamschtein C. Self-Sovereign Patient as a Cornerstone of Healthcare 40. Blockchain Healthc Today. (2025) 8:1. doi: 10.30953/bhty.v8.414 PMC12860432 41623331

[50] Soares A Piçarra N Giger JC Oliveira R Arriaga P. Ethics 4.0: ethical dilemmas in healthcare mediated by social robots. Int J Soc Robot. (2023) 15:807–23. doi: 10.1007/s12369-023-00983-5 37251278 PMC9989998

[51] Kneer M Viehoff J. The hard problem of AI alignment: value forks in moral judgment. proceedings of the 2025. In: *ACM Conference on Fairness, Accountability, and Transparency* (2025). doi: 10.2139/ssrn.5272172

[52] Souza F Souza A Amorim BF Cordeiro T. Towards Role definition in agile ai-based system development: perspectives and reflections. In: Proceedings of the XXIII Brazilian Symposium on Software Quality. New York, NY: Association for Computing Machinery (ACM) (2024). doi: 10.1145/3701625.3701661

[53] Cestonaro C Delicati A Marcante B Caenazzo L Tozzo P. Defining medical liability when artificial intelligence is applied on diagnostic algorithms: a systematic review. Front Med. (2023) 10:1305756. doi: 10.3389/fmed.2023.1305756 38089864 PMC10711067

[54] Goodman K Litewka S Malpani R Pujari S Reis A. Global health and big data: the WHO's artificial intelligence guidance. South Afr J Sci. (2023) 119:14725. doi: 10.17159/sajs.2023/14725 39328373 PMC11426405

[55] European Union. Regulation Laying Down Harmonised Rules on Artificial Intelligence (Artificial Intelligence Act). Official Journal of the European Union (2024).

[56] World Health Organization. Ethics and Governance of Artificial Intelligence for Health: Guidance on Large Multi-Modal Models. Geneva: World Health Organization (2024). Available online at: https://www.who.int/publications/i/item/9789240084759 (Accessed March 27, 2026).

[57] Fahrner LJ Chen E Topol E Rajpurkar P. The generative era of medical AI. Cell. (2025) 188:3648–60. doi: 10.1016/j.cell.2025.05.018 40645169

[58] Kim Y Jeong H Park C Park E Zhang H Liu X . Tiered agentic oversight: a hierarchical multi-agent system for AI safety in healthcare. ArXiv:2506.12482. (2025).

[59] Phiri CC. Creating characteristically auditable agentic AI systems. In: Proceedings of the Intelligent Robotics FAIR (2025). doi: 10.1145/3759355.3759356

[60] Kulothungan V. Using blockchain ledgers to record AI decisions in IoT. IoT. (2025) 6:37. doi: 10.3390/iot6030037

[61] American Medical Association. AMA Adopts Policy to Advance AI Literacy in Medical Education (2025). Available online at: https://www.ama-assn.org/press-center/ama-press-releases/ama-adopts-policy-advance-ai-literacy-medical-education (Accessed March 24, 2026).

[62] American Medical Association. To Implement Health AI, First Decide Who's Accountable (2025). Available online at: https://www.ama-assn.org/practice-management/digital-health/implement-health-ai-first-decide-who-s-accountable (Accessed March 24, 2026).

[63] Esterhuyse CA Müller T van Binsbergen LT. JustAct: actions universally justified by partial dynamic policies. In: Formal Techniques for Distributed Objects Components and Systems. Castiglioni V Francalanza A, editors. Cham: Springer Nature Switzerland (2024). p. 60–81. doi: 10.1007/978-3-031-62645-6_4

